# Design and Rationale for comParison Between ticagreLor and clopidogrEl on mIcrocirculation in Patients with Acute cOronary Syndrome Undergoing Percutaneous Coronary Intervention (PLEIO) Trial

**DOI:** 10.1007/s12265-017-9783-8

**Published:** 2018-01-17

**Authors:** Kyungil Park, Young-Rak Cho, Jong-Sung Park, Tae-Ho Park, Moo-Hyun Kim, Young-Dae Kim

**Affiliations:** 1 Regional Cardiocerebrovascular Center, Division of Cardiology, Department of Internal Medicine, Dong-A University Hospital, Dong-A University College of Medicine, Busan, Republic of Korea; 2 Regional Cardiovascular Center, Division of Cardiology, Department of Internal Medicine, Dong-A University Hospital, Dong-A University College of Medicine, Daesingongwon 26, Seo-gu, Busan, 49201 Republic of Korea

**Keywords:** Ticagrelor, Clopidogrel, Microcirculation

## Abstract

It has been previously demonstrated that ticagrelor can reduce mortality compared to clopidogrel in acute coronary syndrome (ACS) patients. However, the mechanism for this mortality reduction remains uncertain. The objective of the present study is to assess the impact of chronic ticagrelor treatment on microvascular circulation. A total of 120 participants aged 20–85 years with clinical diagnosis of ACS will be randomized in a 1:1 fashion to the following two groups: ticagrelor 90 mg twice daily; clopidogrel 75 mg once daily. To evaluate the status of microcirculation, the primary end point is coronary microvascular dysfunction measured using an index of microcirculatory resistance (IMR) at 6 months after receiving the study agent. The purpose of this trial is to investigate whether ticagrelor, beyond its antiplatelet efficacy, could improve coronary microcirculation more effectively than clopidogrel for patients with ACS.

## Background

Acute coronary syndrome (ACS) is a condition of suddenly reduced blood flow to the heart. Patients with ACS have elevated levels of platelet activity [1, 2]. They are at substantial increased risk for adverse cardiovascular events. Current treatment guidelines recommend antiplatelet therapy to reduce the risk of myocardial infarction, stroke, and cardiac death in patients with ACS [3, 4]. Ticagrelor is a reversible, potent, and oral adenosine diphosphate (ADP) P2Y12 receptor blocker developed as a treatment for patients with ACS. It has been demonstrated that ticagrelor can reduce the mortality of patients with ACS compared to clopidogrel from Platelet Inhibition and Patient Outcomes (PLATO) study [5]. However, the mechanism involved in its mortality reduction remains uncertain.

Several hypotheses have been suggested to explain the mechanism of the impressive benefit of ticagrelor beyond its antiplatelet action. One hypothesis is adenosine theory that some benefits of ticagrelor might be attributed to adenosine-medicated mechanism. Adenosine has been proposed to have cardioprotective effect as a physiologically potent vasodilator [6]. Several clinical studies support the hypothesis that adenosine could reduce cardiac ischaemia reperfusion damage [7, 8]. Ticagrelor can increase levels of extracellular adenosine by inhibiting adenosine reuptake [9, 10], leading to a significant increase in adenosine-induced coronary vasodilatory response [11]. Through these pathways, ticagrelor might be able to prevent microvascular dysfunction. A recent study has reported that in ST-segment elevation myocardial infarction (STEMI) patients, 180 mg loading dose of ticagrelor might be more effective in reducing microvascular injury than 600 mg loading dose of clopidogrel as demonstrated by an index of microcirculatory resistance (IMR) immediately after primary percutaneous coronary intervention (PCI) [12]. However, there are no available data on coronary microcirculation effects after chronic treatment of ticagrelor in patients with ACS who have altered resting coronary blood flow dynamics due to advanced coronary artery disease.

Therefore, the objective of this comparison trial of ticagrelor and clopidogrel on microcirculation in patients with ACS undergoing PCI is to determine whether ticagrelor might have potential benefit on coronary microcirculation in ACS patients undergoing PCI during 6 months follow-up period compared to clopidogrel.

## Methods/Design

### Study End Points

The primary hypothesis to be tested is whether ticagrelor could significantly improve coronary microcirculation in ACS patients undergoing PCI with 6 months of follow-up period compared to clopidogrel. To evaluate the status of microcirculation, the primary end point is coronary microvascular dysfunction measured using an IMR at 6 months after receiving the study agent. Secondary end points include fractional flow reserve (FFR), coronary flow reserve (CFR), and a change in IMR from randomization to the end of treatment using coronary pressure-temperature wire on culprit lesion during 6 months of follow-up.

### Participants

Patients are eligible for enrollment if they are hospitalized for ACS with or without ST-segment elevation with an onset of symptoms during previous 24 h. All patients with ACS scheduled for PCI are eligible if they could understand the character and individual consequences of participation and provide written informed consent.

Exclusion criteria are (1) less than 20 years or more than 85 years old, (2) the presence of hemodynamic instability, (3) intolerance to antiplatelet drugs, (4) ineligible for coronary revascularization, (5) liver cirrhosis greater than or equal to Child class B, (6) decreased serum platelet level (< 100,000/uL), (7) need chronic oral anticoagulant therapy, (8) ACS in previous month, (9) a history of myocardial infarction in the target vessel-related territory, (10) a history of coronary artery bypass grafts, (11) renal dysfunction with creatinine > 3 mg/dL, (12) chronic total occlusion, (13) in-stent restenosis, (14) left main lesion, (15) contraindications to adenosine, (16) previous treatment with statins, (17) a life expectancy < 1 year, and (18) an inability to give informed consent. Complete listings of our inclusion and exclusion criteria are provided in Table 1.

**Table 1:** Inclusion and exclusion criteria

Inclusion criteria
Hospitalized for potential ST-segment elevation or non-ST-segment elevation ACS, with onset during the previous 24 h, documented by cardiac ischemic symptoms due to atherosclerosis of ≥ 10 min’ duration at rest, male and female aged 20 to 85 years, not pregnant, and with informed consent And at least one of the following: 1) ST-segment changes on ECG indicating ischemia. ST-segment depression or transient elevation ≥ 1 mm in two or more two contiguous leads 2) Positive biomarker indicating myocardial necrosis. Troponin-I or CK-MB greater than the upper limit of normal or persistent ST-segment elevation ≥ 1 mm (not known to be preexisting or due to a coexisting disorder) in ≥ 2 contiguous leads or new LBBB plus primary PCI planned.
Exclusion criteria
1) Less than 20 years or more than 85 years old 2) The presence of hemodynamic instability 3) Intolerance to antiplatelet drugs 4) Ineligible for coronary revascularization 5) Liver cirrhosis greater than or equal to Child class B 6) Decreased serum platelet level (< 100,000/uL) 7) Need chronic oral anticoagulant therapy 8) Acute coronary syndrome in previous month 9) History of myocardial infarction in the target vessel-related territory 10) History of coronary artery bypass grafts 11) Renal dysfunction with creatinine > 3 mg/dL 12) Chronic total occlusion 13) In-stent restenosis 14) Left main lesion 15) Contraindications to adenosine 16) Previous treatment with statins 17) A life expectancy < 1 year 18) An inability to give informed consent.

*ACS* acute coronary syndrome, *ECG* electrocardiogram, *LBBB* left bundle branch block

### Study Protocol

This trial is designed as a non-blinded, open label, parallel-group, prospective, and randomized controlled clinical trial performed in a single center. Written informed consent is obtained from each participant before diagnostic coronary angiogram and randomization. After baseline measurement, participants are randomly allocated into ticagrelor or clopidogrel group. All patients are pretreated with aspirin 300 mg orally and a loading dose of ticagrelor 180 mg or clopidogrel 600 mg according to the randomization. After PCI for index vessel, physiologic measures including FFR, CFR, and IMR are obtained twice (6 months apart) from the culprit artery after PCI. Patients will receive ticagrelor 90 mg twice daily or clopidogrel 75 mg once daily for at least 6 months. All patients in PLEIO trial are treated with rosuvastatin 5 mg (Crestor, AstraZeneca, Cambridge, UK) per day during the study period. Any patient who is found to take less than 80% or more than 120% of the assigned study drug is considered non-compliant as assessed by tablet counts [13]. All patients are advised to maintain aspirin (≥ 80 mg/day) lifelong. The trial is overseen by an independent data monitoring committee.

**Fig. 1:**
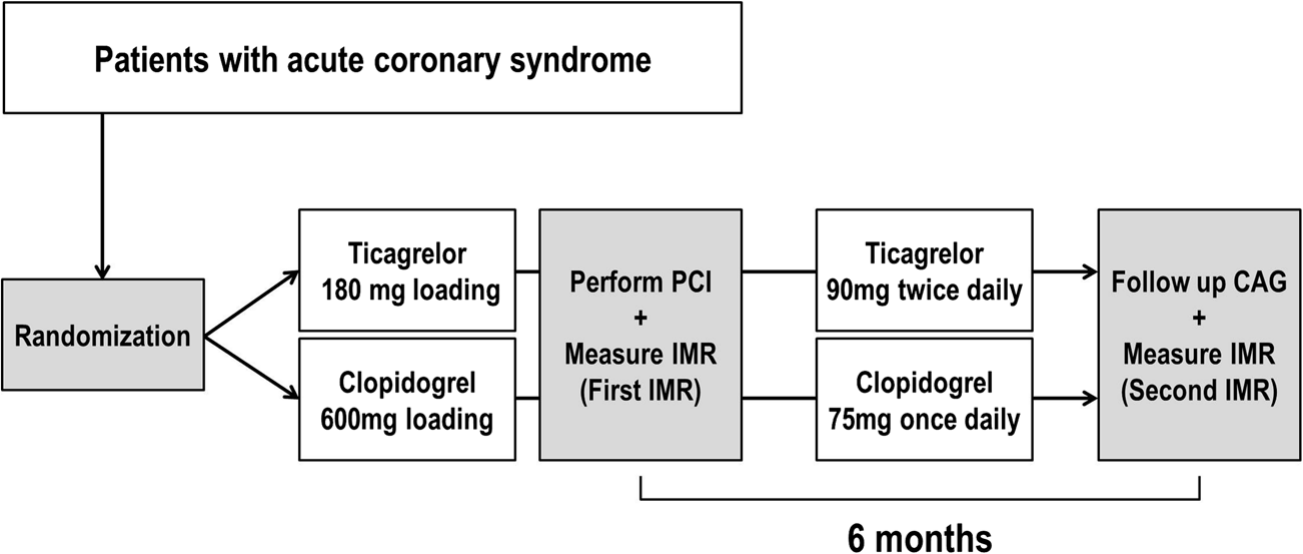
PLEIO study design. PCI percutaneous coronary intervention, IMR index of microcirculatory resistance, CAG coronary angiogram

Recruitment of participants was started in December 2014. The total number of randomized subjects will be 120. Baseline characteristics of these patients enrolled in PLEIO are shown in Table 2. The process of the trial conduct is illustrated in Fig. 1. It was registered under www.clinicaltrials.gov: NCT02618733.

**Table 2:** Clinical characteristics and treatment at baseline

	Ticagrelor (*n* = 60)	Clopidogrel (*n* = 60)	*p* value
Age	56.9 ± 11.4	58.5 ± 9.9	0.14
Male	46 (76.7%)	44 (73.3%)	0.69
Height (cm)	167.4 ± 8.1	165.5 ± 7.7	0.20
Weight (kg)	70.5 ± 12.8	66.4 ± 10.6	0.06
Risk factors			
Diabetes	21 (35.0%)	22 (36.7%)	0.50
Hypertension	27 (45.0%)	27 (45.0%)	0.57
Hypercholesterolemia	18 (30.0%)	17 (28.3%)	0.50
Current smoker	35 (59.3%)	35 (42.4%)	0.09
Ejection fraction (%)	52.0 ± 8.7	52.4 ± 8.9	0.81
Biomarker			
Serum hemoglobin (g/dL)	14.5 ± 1.7	14.0 ± 1.4	0.07
Platelet count (× 10^9^/L)	23.9 ± 5.5	22.9 ± 6.9	0.40
Serum creatinine (mg/dL)	1.0 ± 0.3	1.0 ± 0.2	0.15
HbA1c (%)	6.7 ± 1.7	6.3 ± 1.3	0.10
Lipid profile			
Total cholesterol	184.3 ± 45.1	195.8 ± 45.8	0.17
LDL cholesterol	119.5 ± 36.4	115.5 ± 32.3	0.53
HDL cholesterol	41.9 ± 9.2	44.1 ± 11.9	0.27
Triglycerides	182.5 ± 87.3	186.1 ± 72.8	0.23
Cardiac troponin-I	49.1 ± 58.9	39.9 ± 70.8	0.44
Brain natriuretic peptide	87.9 ± 161.9	124.8 ± 216.7	0.33
Clinical diagnosis			0.51
STEMI	18 (30.0%)	20 (33.3%)	
NSTEMI	29 (48.3%)	23 (38.3%)	
Unstable angina	13 (21.7%)	17 (28.3%)	
Platelet function, 6 months			
Platelet reactivity (PRU)	44.3 ± 47.3	187.4 ± 71.1	< 0.001
% Inhibition	84.3 ± 16.4	27.9 ± 21.9	< 0.001
Target vessel			0.53
Left anterior descending artery	29 (48.3%)	30 (50%)	
Left circumflex artery	15 (25.0%)	13 (21.7%)	
Right coronary artery	16 (26.7%)	17 (28.3%)	
Number of vessels diseased,(*n*)			0.74
1-vessel disease	41 (68.3%)	37 (61.7%)	
2-vessel disease	16 (26.7%)	19 (31.7%)	
3-vessel disease	3 (5.0%)	4 (6.7%)	
ACC lesion type			0.34
A	6 (10.0%)	2 (3.3%)	
B1	17 (28.3%)	13 (21.7%)	
B2	26 (43.3%)	32 (53.3%)	
C	11 (18.3%)	13 (21.7%)	
Stents (*n*)	1.0 ± 0.2	1.0 ± 0.3	0.70
Stent diameter (mm)	2.9 ± 0.5	2.9 ± 0.4	0.32
Stent length (mm)	23.6 ± 8.9	24.4 ± 10.5	0.66

*STEMI* ST-segment elevation myocardial infarction, *NSTEMI* non-ST-segment elevation myocardial infarction

### Randomization

Random assignments are generated using the Excel spreadsheet software (Microsoft Corporation, Redmont, CA, USA). Eligible patients are randomly assigned in a 1:1 ratio to receive treatment with ticagrelor or clopidogrel. Randomization is performed by a person not involved in this study and kept concealed. Ticagrelor will be given in a loading dose of 180 mg followed by a dose of 90 mg twice daily. Patients in the clopidogrel group will receive 600 mg loading dose followed by a dose of 75 mg once daily. The randomized treatment will be continued for 6 months.

### Coronary Intervention and Physiologic Measurements

PCI is performed according to the latest standard guidelines [14, 15]. All culprit lesions are treated with biodegradable polymer drug-eluting stent (Orsiro, Biotronik, Berlin, Germany). Angiographic success is defined as a residual stenosis ≤ 30% by visual analysis in the presence of Thrombolysis In Myocardial Infarction (TIMI) grade 3 flow.

Physiologic measurements are performed for the culprit vessel immediately and after 6 months after PCI. A six to seven French coronary guiding catheter without side-hole is used to engage the selected coronary artery. An intracoronary pressure and temperature sensor-tipped guidewire (PressureWire Certus, ST. Jude Medical, MN, USA) is used to measure distal coronary pressure. The index of coronary flow is derived from the coronary thermodilution method as described previously [16]. After the culprit lesion is successfully treated, the guidewire is calibrated for pressure recording. It is then equalized with aortic pressure in the guiding catheter after injection of 200 μg of intracoronary nitroglycerin. The tip pressure sensor is advanced across the stented segment and beyond the mid-to-distal portion of the culprit vessel. Aortic pressure (Pa) and distal intracoronary pressure (Pd) are obtained at baseline. FFR is calculated as the mean Pd divided by mean Pa during maximal hyperemia. To measure CFR, the coronary flow under basal conditions is determined by intracoronary administration of 3 mL of room temperature saline three times in succession (3 mL/s) manually. Maximal hyperemia is then induced, and three additional intracoronary room temperature saline boluses of 3 mL are administered to determine peak coronary flow presented as peak mean transit time (Tmn). CFR is calculated based on the ratio of mean transit times during hyperemia and at baseline. After evaluation of FFR and CFR, IMR is calculated. In culprit vessel, a simplified method of IMR is calculated as follows:

$$ \mathrm{IMR}=\mathrm{Pd}\ \mathrm{during}\  \mathrm{maximal}\  \mathrm{hyperemia}\times \mathrm{Tmn} $$

To induce maximal hyperemia, intravenous adenosine is administered via the right femoral vein (140 mg/kg/min).

### Platelet Function Measurements

Platelet function measurements are performed to assess platelet reactivity. Blood sample withdrawal for assessing platelet function is conducted at least 6 h after the loading dose of clopidogrel or 2 h after the loading dose of ticagrelor [17]. Blood samples are acquired via sheath in the catheterization laboratory prior to each physiologic measurement and collected in 3.2% citrate Vacuette tubes (Greiner Bio-One Vacuette North America, Inc., Monroe, NC, USA). Platelet reactivity is measured using VerifyNow P2Y12 assay (Accumetrics, San Diego, CA, USA). It is numerically expressed in P2Y12 reaction units (PRU). The time interval between blood sampling and VerifyNow P2Y12 testing does not exceed 2 h.

### Quality of Life Measurements

Quality of life (QOL) is assessed by using the Korean version of the Medical Outcomes Study 36-Item Short-Form Health Survey (SF-36) questionnaire at first day and 6 months after PCI. Data from the QOL questionnaire are self-reported by patients in the presence of the study psychologist. A license for the use of SF-36 v.2 was obtained from Optuminsight Life Sciences, Inc. (license number QM027885).

### Clinical End Points

Clinical follow-up of patients will be conducted in-hospital, at 4 weeks, 6 months, and 12 months after PCI. In PLEIO, clinical end points are bleeding events and cardiovascular events. Bleeding is measured using the Bleeding Academic Research Consortium (BARC) definition [18]. Bleeding events are further classified based on bleeding severity according the Global Use of Strategies to Open Occluded Coronary Arteries (GUSTO) bleeding criteria and TIMI [19, 20]. Cardiovascular events are defined as cardiovascular death, myocardial infarction, clinically driven target lesion revascularization, stent thrombosis, stroke, or non-target lesion revascularization [21].

### Sample Size

Sample size calculation was based on primary end point and primary analysis for the intention-to-treat population. Sample size was calculated using the study’s primary objective to detect a 30% relative difference in IMR measurements between ticagrelor and clopidogrel treatment with a power of 90% to demonstrate difference. On the basis of previous reports, we assumed the value of IMR after PCI to be approximately 35 U in ACS patients with clopidogrel treatment [12, 22, 23]. We expected that the relative difference in IMR measurement would be at least 30% when considering ACS patients. The assumed result of IMR after ticagrelor treatment was 25 U based on observations made in a pilot study performed to inform the design of the current trial. We adjusted the sample size considering an estimated follow-up loss rate of 20% with a two-sided level of significance *α* = 5% and a power of 1-β = 90%, resulting in 60 patients in each group to detect this difference with a two-sided Student’s *t* test. Therefore, a total of 120 patients will be randomized and included in this study.

### Statistical Analysis

Continuous data are analyzed using Student’s *t* test. Chi-squared test is used to analyze categorical variables. The focus of this trial is to compare the efficacy by measuring IMR between the two study groups. Statistical analyses are performed on an intention-to-treat basis. The two-sided null hypothesis for the primary end point states is that ticagrelor has potential benefit to improve coronary microcirculation in ACS patients undergoing PCI. Primary end point is assessed using Student’s *t* test if samples are normally distributed or their variances are homogeneous. Otherwise Mann-Whitney *U* test is used. Two-way analysis of variance (ANOVA) with repeated measures is used to detect changes in IMR values from randomization to the end of treatment in the two study groups. The operator’s reproducibility is evaluated by using both linear regression analysis and the Bland-Altman method to assess limits of agreement between repeated measurements.

Safety analysis includes calculation of frequencies and rates of bleeding reported in the two groups. This analysis is performed on the full analysis set which consisted of patients who received at least one dose of the study medication. Graphical methods including scatter plots and boxplots are used to visualize findings of the trial. The Kaplan-Meier method is used to plot the time to the first episode of cardiovascular events after PCI. Subgroup analyses will be performed to evaluate QOL among ACS patients. Difference in QOL between hospital admission and 6-month follow-up will be categorized as improvement in QOL when the difference is greater than zero or lack of improvement when the difference is less than zero. All statistical analyses are performed using SPSS version 16.0 or higher. Statistical significance is considered at *p* < 0.05.

## Discussion

We hypothesized that ticagrelor, beyond its antiplatelet efficacy, could improve coronary microcirculation more effectively than clopidogrel for patients with ACS, resulting in a reduction of mortality.

### The Mechanism of Mortality Benefit with Ticagrelor

Ticagrelor has shown promising results in clinical trials [5, 24, 25]. It is now preferred over clopidogrel for management of patients with ACS who undergo stent implantation and those who are managed with medical therapy [3, 4]. In the PLATO study, compared to clopidogrel, ticagrelor has impressive mortality benefit for patients with ACS [5]. Although the mechanism of this result is not fully understood, several hypotheses have been suggested. One hypothesis is that more potent and consistent antiplatelet effect of ticagrelor might have contributed to the lower incidence of cardiovascular death [26, 27]. Platelet activation and aggravation are key factors in the development of ACS. Thus, platelets are considered principal therapeutic targets in the management of ACS [1, 3, 4, 28]. Ticagrelor has greater antiplatelet effects with reversibility compared to clopidogrel [29]. Greater platelet inhibition has been associated with improved myocardial perfusion before and after PCI. Improvements in myocardial perfusion in turn are associated with improved clinical outcomes [30]. Reversibility allows redistribution of ticagrelor to all platelets in circulation. Redistribution of ticagrelor is expected to attenuate the reactivity of new platelets more consistently and effectively than clopidogrel. Accordingly, ticagrelor contributes to more consistent antiplatelet effect than clopidogrel. However, it may be difficult to explain the benefit of ticagrelor by its antiplatelet properties [31] because prasugrel is not associated with significant reduction in cardiovascular mortality although it has greater antiplatelet effects compared to clopidogrel [32].

Another suggested hypothesis is that increased blood level or enhanced effects of adenosine by ticagrelor might contribute to its mortality reduction benefit. Adenosine is an endogenous nucleoside found in large quantities in myocardial and endothelial cells [33, 34]. It is known to regulate myocardial and coronary circulatory functions. Its plasma levels are increased after cellular stresses such as ischemia and inflammation [35]. It activates four well-characterized receptors, producing various physiological effects [36, 37]. Accordingly, adenosine modulates coronary vasodilation and inflammatory responses to a variety of stressful condition [38]. In addition, it has been reported that adenosine can reduce ischemia/reperfusion injury [39]. Moreover, adenosine may prevent microvascular spasm and modulate endothelial damage of coronary microcirculation [40, 41]. Plasma concentration of adenosine might have increased, because ticagrelor is a precursor of adenosine that can inhibit cellular uptake of adenosine [9, 10]. Increased adenosine release may exert its biological effects. A recent animal study has shown that ticagrelor can reduce infarct size to a significantly greater extent than clopidogrel and this might be associated with its adenosine-dependent mechanism [42]. A recent clinical trial has shown that 180 mg loading dose of ticagrelor has microvascular protective potential in STEMI patients [12]. This effect may contribute to its mortality reduction benefit compared to clopidogrel.

### Microvascular Dysfunction in ACS Patients

ACS is the leading cause of morbidity and mortality [2] in coronary artery disease. To predict clinical outcomes in patients with ACS, it is important to assess myocardial viability, for which microvascular function and integrity are the most important determinants. Coronary microcirculation which consists of resistance arterioles, capillaries, and small veins plays a major part in the delivery of blood and nutrients to the myocardium [43]. Microvascular dysfunction represents a predisposing factor for myocardial ischemia [44]. It is also an important prognostic factor of ACS [45, 46]. Despite the importance of microvascular coronary damage for prognosis, an accurate evaluation of microvascular function is challenging. Currently, no technique allows direct visualization of coronary microcirculation in vivo in humans. Most parameters for evaluating microvascular function rely on quantification of coronary blood flow. Among them, TIMI myocardial perfusion grade is a simple and useful clinical tool for microcirculation assessment. Even though ticagrelor has survival benefit, angiographic analysis from PLATO trial has shown that there is no significant difference in myocardial perfusion grade between ticagrelor and clopidogrel treatment groups [47]. Recently, IMR has been proposed and validated for assessing the status of microcirculation. Technological advance in measuring pressure and estimating coronary artery flow simultaneously using a single pressure-temperature sensor-tipped coronary wire has made it possible. IMR provides a quantitative measure of coronary microvasculature status. It is independently associated with LV function and infarct pathology [48, 49]. However, the protocol of the PLATO study did not involve IMR measurements or non-invasive tools such as positron emission tomography or magnetic resonance. Therefore, effects of ticagrelor maintain therapy on the prevention of microvascular dysfunction in ACS patients remain uncertain.

### PLEIO Study

The PLEIO trial is conducted to test the hypothesis that ticagrelor could improve coronary microcirculation (estimated by IMR) more effectively than clopidogrel for patients with ACS during 6 months of follow-up period. This is a short-term clinical study. However, it is the first trial investigating the potential benefits of6 months of ticagrelor treatment on coronary microcirculation in ACS patients undergoing PCI. As of September 2017, there are no registered trials evaluating therapeutic potentials of 6 months of ticagrelor treatment in preventing microvascular dysfunction. If potential benefits of ticagrelor on coronary microcirculation are proved, results of the present study may provide a potential mechanism for mortality reduction compared to clopidogrel.
